# Susceptibility to advanced age-related macular degeneration and alleles of complement factor H, complement factor B, complement component 2, complement component 3, and age-related maculopathy susceptibility 2 genes in a Mexican population

**Published:** 2012-10-11

**Authors:** Beatriz Buentello-Volante, Gabriela Rodriguez-Ruiz, Antonio Miranda-Duarte, Ericka N. Pompa-Mera, Federico Graue-Wiechers, Carolina Bekker-Méndez, Raul Ayala-Ramirez, Carlos Quezada, Jose L. Rodríguez-Loaiza, Juan C. Zenteno

**Affiliations:** 1 Department of Genetics and Research Unit, Institute of Ophthalmology “Conde de Valenciana” Mexico City, Mexico; 2 Department of Genetics, National Rehabilitation Institute, Mexico City, Mexico; 3 Unidad de Investigación Médica en Inmunología e Infectología, Hospital de Infectología, Centro Médico Nacional La Raza, IMSS, Mexico City, Mexico; 4 Retina Department, Institute of Ophthalmology “Conde de Valenciana” Mexico City, Mexico; 5 Faculty of Medicine, Department of Biochemistry, National Autonomous University of Mexico, Mexico City, Mexico

## Abstract

**Purpose:**

To investigate the association of age-related macular degeneration (AMD)–high risk alleles of the complement factor H (*CFH*), complement factor B (*CFB*), complement component 2 (*C2*), complement component 3 (*C3*), and age-related maculopathy susceptibility 2 (*ARMS2*) genes in a Mexican population for the first time.

**Methods:**

Genotyping was performed for the Y402H variant of CFH, for the L9H, R32Q, and K565E variants of CFB, the E318D variant of C2, the A69S variant of ARMS2, and the R102G variant of C3 in 159 Mexican mestizo patients at advanced stages of AMD, i.e., CARMS (Clinical Age-Related Maculopathy Staging System) grade 4 or 5. The frequency of these variants was also investigated in a group of 152 control subjects without AMD. Genomic DNA was extracted from blood leukocytes, and genotyping was performed using PCR followed by direct sequencing. Allele-specific restriction enzyme digestion was used to detect the R102G polymorphism in C3.

**Results:**

There were significant differences in the allelic distribution between the two groups for CFH Y402H (p=1×10^−5^), ARMS A69S (p=4×10^−7^), and CFB R32Q (p=0.01). The odds ratios (95% confidence interval) obtained for the risk alleles of these three variants were 3.8 (2.4–5.9), 3.04 (2.2–4.3), and 2.5 (1.1–5.7), respectively. Haplotype analysis including the two most significantly associated alleles (CFH Y402H and ARMS A69S) indicated that the C-T combination conferred an odds ratio (95% confidence interval) of 6.9 (3.2–14.8). The exposed attributable risk for this particular haplotype was 85.5%.

**Conclusions:**

This is the first case-control investigation of AMD–high risk alleles in a Latino population. Our results support that *CFH*, *ARMS2*, and *CFB* AMD-risk alleles are consistently associated with the disease, even in ethnic groups with a complex admixture of ancestral populations such as Mexican mestizos.

## Introduction

Age-related macular degeneration (AMD) is a progressive, neurodegenerative retinal disease causing irreversible central vision loss. AMD is the leading cause of legal blindness in the elderly population [1], as approximately one in four individuals aged 75 or older has some sign of this disease and about one in 15 has the advanced form with visual loss [2]. Due to increasing life expectancy, the prevalence of AMD is predicted to grow by more than 50% by 2020, substantially augmenting the health burden of AMD [1].

The early stage of AMD is characterized by drusen formation—white-yellow deposits in Bruch’s membrane under the retinal pigment epithelial (RPE) layer and photoreceptor cells (reviewed in [3]). Advanced AMD can clinically develop in two distinct forms: dry (atrophic) and wet (exudative, neovascular). The slowly progressing and more frequent dry AMD is characterized by the presence of an irregular area of depigmentation as a result of the loss of RPE cells, and causes gradual geographic atrophy of the retina. Wet AMD, responsible for the majority of legal blindness in AMD, is characterized by choroidal neovascularization with leakage and bleeding, leading to the irreversible damage of photoreceptors (reviewed in [4]).

AMD is a multifactorially determined disease in which the interplay of environmental and genetic factors causes the disorder. Advanced age is by far the major nongenetic factor [5-7], although traits as smoking [8], gender [9], body mass index [9,10], ethnicity [10], and a high-fat diet [11] have also been implicated as factors affecting AMD risk.

Genetic factors play a role in disease development, as indicated by twin studies and recurrence risks in first-degree relatives of patients with AMD [12,13]. The disease has recurrence ratios for siblings of a case that are 3–6-fold higher than in the general population [14], and estimates of the genetic hereditability of AMD range up to 71% [12,15]. However, the genetic variants known to date are estimated to account for <50% of the heritability of the disease [16,17].

Genome-wide association studies first revealed the association between polymorphisms in the complement factor H (*CFH*) gene and susceptibility to AMD [18,19]. Since then, risk-associated polymorphisms in complement factor I (*CFI*) [20], complement factor B (*CFB*) [21], complement component 2 (*C2*) [21], complement component 3 (*C3*) [22], complement factor H-related 3 (*CFHR3*), complement factor H-related 1 (*CFHR1*) [23], and age-related maculopathy susceptibility 2 (*ARMS2*) [24] genes have been discovered and replicated in several ethnic groups worldwide (for recent reviews, see [25-27]). Three allelic variants—CFH Y402H, ARMS2 A69S, and C3 R102G—account for approximately 76% of the population-attributable risk of the development of AMD [26].

CFH is a serum glycoprotein that regulates the function of the alternative complement pathway. CFH binds to C3b, accelerates the decay of the alternative pathway convertase C3bBb, and acts as a cofactor for complement factor I, another C3b inhibitor [28,29]. The CFB, C2, and C3 genes encode proteins that play central roles in activating classical and alternative complement pathway systems. The *ARMS2* gene (previously known as LOC387715) encodes a 107–amino acid protein of unknown function with nine predicted phosphorylation sites; this gene is expressed in the retina and in various other tissues.

To date, most of the studies reporting the association between such genetic variants and AMD risk have been undertaken in people of Caucasian and Asian origin [30]. Conversely, there are none or very few studies on populations from the African continent or indeed from other regions, such as the Middle East, parts of Asia, and South America [30]. Independent replication of disease-associated alleles in populations that are not traditionally screened is important for further delineation of the molecular basis of AMD and for the possible identification of ethnic-specific differences in the magnitude with which particular genetic variants modify disease risk. Up to now, the association between AMD risk and genetic polymorphisms has not been investigated in Latino populations, which are ethnic groups with a considerable genetic admixture. The purpose of this study is to present the results of the first association study between AMD and complement-related gene polymorphisms in a Mexican population.

## Methods

One hundred and fifty-nine nonfamilial patients with advanced AMD and 152 normal controls were recruited following a standard ophthalmologic examination protocol. This investigation was a hospital-based, case-control association study undertaken in a Mexican population. The study was approved by the Institutional Review Board of the Institute of Ophthalmology “Conde de Valenciana,” Mexico City. Informed consent was signed by all subjects before they participated in the study. Ophthalmic records, stereo fundus photographs, and fluorescein angiograms were obtained for all patients. Grading was performed using the Clinical Age-Related Maculopathy Staging System (CARMS) classification [5,31]. All the participants were of Mexican mestizo origin. A Mexican mestizo is defined as a person who was born in the country, has a Spanish-derived last name, and has a family of Mexican ancestors back to the third generation [32].

Criteria for patient inclusion were as follows: (1) age 55 years or older, (2) diagnosis by a retina specialist of AMD grades 4 or 5 in both eyes or AMD grades 4 or 5 in one eye and any type of drusen in the fellow eye, (3) no association with other retinal disease, and (4) a negative history of vitreoretinal surgery. CARMS grade 4 corresponds to geographic atrophy, while grade 5 corresponds to choroidal neovascularization. The AMD stage assigned was based on the most severe eye at the time of recruitment. Control subjects—persons without visual impairment—were recruited from the outpatient department during routine ophthalmic examination. They were aged 60 years or older, had no drusen or RPE changes under dilated fundus examination, and reported a negative family history of AMD.

### Single nucleotide polymorphism genotyping

The regions of the *CFH* gene harboring the single nucleotide polymorphisms (SNPs) rs1061170 (Y402H); the *CFB* gene harboring rs4151667 (L9H), rs641153 (R32Q), and rs4151659 (K565E); the *C2* gene harboring rs9332739 (E318D); the *ARMS2* gene including rs10490924 (A69S); and the *C3* gene carrying rs2230199 (R102G) were amplified in independent PCR reactions. Oligonucleotide sequences are available in Table 1. Each 25 μl PCR amplification reaction contained 1X buffer, 200 ng of genomic DNA, 0.2 mM of each deoxyribonucleotide triphosphate (dNTP), 2U *Taq* polymerase, 1 mM of forward and reverse primers, and 1.5 mM MgCl_2_. PCR products were analyzed in 1.5% agarose gels, from which the bands with the amplified templates were excised and the DNA subsequently purified with the help of the Qiaex II kit (Qiagen, Hilden, Germany). Genotyping of the variants (except R102G) was performed with direct automated sequencing with the BigDye Terminator Cycle Sequencing kit (Applied Biosystems, Foster City, CA), and using a temperature program that included 25 cycles of denaturation at 97 °C for 30 s, annealing at 50 °C for 15 s, and extension at 60 °C for 4 min. Samples were analyzed in an ABI Prism 3130 Genetic Analyzer (Applied Biosystems). SNP rs2230199 (R102G) of the *C3* gene was analyzed with a polymerase chain reaction-restriction fragment length polymorphism (PCR-RFLP) approach using the HhaI restriction enzyme. The rs2230199 PCR-RFLP analysis was verified with direct nucleotide sequencing of several PCR samples of each genotype.

**Table 1:** Oligonucleotide sequences, amplicon sizes, and annealing temperatures for PCR amplification of AMD-related variants.

Amplicon	Forward primer (5′-3′)	Reverse primer (5′-3′)	Size PCR product (bp)	Temperature (°C)
CFH Y402H	AGTTCGTCTTCAGTTATAC	TGGTCTGCGCTTTTGGAAGAGC	263	59.5
CFB L9H	ACACCATCCTGCCCCAGGCCCA	TTGATCTCTACCCCCTCCAG	267	67.2
CFB R32Q	AGCCCCCAACTCTGCCTGATGCCC	GTACTCCAGTGCCTGGCCCTC	252	65.2
CFB K565E	GCGGGACCTGGAGATAGAAGT	TAGTCTGGCCATATTTCAGC	140	59.1
C2 E318D	GTGAGCGTTGCCATTATCACCT	TGAGAGGGTCCATCTTCTC	171	57.6
C2 A69S	CACTCTGCGAGAGTCTGTGCTGGACC	TTTCTTACCAGTGTCAGGTGG	201	65.4
C3 R102G	GATCTCTTTGCCTCTCCTAA	TCTCCTCTTCTGAACTCCTC	720	56.6

### Statistical analysis

Comparisons of continuous variables were tested with the Student *t* test, and corrected chi-square statistics were applied for categorical variables. Uni- and multivariate nonconditional logistic regressions were conducted to determine risk magnitude, comparing each allele and genotype with the main effect employed as the binary variable. Odds ratios (ORs) and 95% confidence intervals (95% CIs) were reported, and population and exposed attributable risks were calculated when the ORs were elevated. The alpha level was 0.05, and the STATA ver. 10.0 statistical software package was used for calculations. Allele frequencies, Hardy–Weinberg equilibrium (H-WE), and haplotype association analysis were assessed with Haplo View 4.0 software (Daly Lab, Broad Institute, Cambridge, MA). For haplotype construction, all genotyped alleles were tested, except those showing H-WE deviation. Only haplotypes that showed statistical significant differences were reported.

## Results

A total of 311 individuals (159 patients with AMD and 152 controls) were genotyped. The mean age at recruitment was 76.4±8.1 years in cases and 73.5±6.8 years in controls (ranges of ages were 60–96 years for cases and 62–90 years for controls). The gender distribution between cases and controls was not significantly different (p=0.8), while smoking frequency showed statistically significant differences between the groups (p=0.04). These and other demographic variables are shown in Table 2.

**Table 2:** Demographic characteristics of patients with age related macular degeneration and controls.

Variable	Cases n=159	Controls n=152	OR (CI 95%)	P value
Age (mean±SD, years)	76.4±8.1	73.5±6.8	1.05 (1.01–1.08)	0.001
Gender (Females, n %)	111 (69.8)	105 (69.1)	1.03 (0.6–1.7)	0.8
Smoking (n %)	37 (23.3)	12 (12.9)	2.04 (1.01–4.2)	0.04
Diabetes mellitus (n %)	29 (20.0)	37 (35.9)	0.4 (0.2–0.8)	0.005
Hypertension (n %)	83 (54.9)	60 (50.4)	1.2 (0.7–1.9)	0.4

OR, odds ratio; CI, confidence interval.

### Association analysis with individual single nucleotide polymorphisms

The allelic and genotypic distributions of the AMD-associated SNPs are shown in Appendix 1. All genotypic distributions in the control group showed H-WE, except variants R32Q and K565E in *CFB* (p<0.05).

Allelic association analysis indicated that there were significant differences in the allelic distribution between the two groups for CFH Y402H, ARMS2 A69S, and CFB R32Q (p=1×10^−5^, 4×10^−7^, and 0.01, respectively). For these variants, the ORs (95% CIs) were as high as 3.8 (2.4–5.9), 3.04 (2.2–4.3), and 2.5 (1.1–5.7), respectively. However, as mentioned above, CFB R32Q was not present in H-WE (Appendix 1). Alleles L9H and K565E of CFB and E318D of C2 did not show significant differences (Appendix 1). The C3 R102G allele showed a tendency to increase the AMD risk; nevertheless, this did not reach statistical significance (OR [95% CI] 1.6 [0.9–2.6]).

Genotypic distributions of CFH Y402H and ARMS2 A69S showed the highest statistically significant differences (Appendix 1). Statistically significant differences were preserved when genotypic frequencies for CFH Y402H and ARMS2 A69S were analyzed adjusting for age, gender, and smoking (Appendix 2).

### Haplotypes

Haplotype analysis, including the two most significantly associated alleles in this study (CFH Y402H and ARMS2 A69S), indicated that the C-T combination conferred an OR (95% CI) of 6.9 (3.2–14.8), followed by the C-G haplotype, which yielded an OR of 2.0 (1.2–3.4). As shown in Table 3, other haplotypes for these variants were also significantly associated with AMD.

**Table 3:** Haplotype analysis results of Complement Factor H (CFH) and Age-Related Maculopathy Susceptibility 2 (ARMS2) in AMD for Mexican cases and controls.

Haplotype		Case, control frequencies	Chi Square	P value	OR (95%CI)	A-e%	A-p%
CFH	ARMS2						
Y402H	A69S						
T	G	0.339, 0.660	64.059	1.2×10^−15^	0.3 (0.2–0.4)		
T	T	0.368, 0.241	11.842	6.0×10^−4^	1.8 (1.3–2.6)	44.4	16.1
C	G	0.136, 0.073	6.405	0.01	2.0 (1.2–3.4)	50	6.5
C	T	0.157, 0.025	31.992	1.5×10^−8^	6.9 (3.2–14.8)	85.5	10.5

OR, odds ratio; CI, confidence interval; A-e%: attributable risk among exposed; A-p%: population attributable risk

### Attributable risk percent

Attributable risk percentages among exposed (Ae%) were calculated for the CFH-ARMS2 risk haplotype (C-T). The population attributable risk was 10.5%, while the exposed attributable risk was 85.5%. Other haplotypes for these alleles were also associated with a high attributable risk (Table 3).

## Discussion

AMD is estimated to affect about 50 million people worldwide [33,34], and an increase in aging populations makes this degenerative disease a significant public health concern. It has been recognized for a long time that AMD is a complex disease with environmental and genetic factors interacting to allow for its development. The strongest identifiable risk factors for AMD are age, family history, genetics, and smoking [25].

In recent years, great advances have been made in recognizing several genetic factors predisposing individuals to AMD. Among these, polymorphisms in several proteins such as C3, FB, and CFH have been demonstrated to strongly influence the risk for AMD with quoted ORs for homozygotes as high as 3.51–7.4 for CFH Y402H [18,35] and 2.6 for C3 R102G [36]. Remarkably, three SNPs-coding for Y402H, ARMS2 A69S, and C3 R80G—account for approximately 76% of population attributable risk of the development of AMD [26,36]. Except ARMS2 A69S, these variants are located in complement alternative pathway proteins.

Although these polymorphic alleles have been unquestionably associated with AMD in patients from distinct ethnic backgrounds, independent replication studies are required to accurately assess these alleles’ ethnospecific contribution to this complex disease. In this study, we genotyped seven common AMD risk-associated SNPs in a cohort of 311 Mexican individuals, 159 of whom had advanced stages of the disease. To the best of our knowledge, this is the first evaluation of the contribution of AMD-related polymorphisms in a Latin American population. The Latin American population is composed mainly of a mix of indigenous people, Africans, and Spaniards (or Portuguese), so it represents an interesting genetic pool to compare the effect of risk alleles, which have been previously characterized in Caucasian, Asian, and African populations. In particular, Mexican mestizos are the result of the continuous intermixing between the autochthonous Mexicans and the Spaniards, who came to America during the 16th century. Mexicans have an average of 52% Native American ancestry, 45% European ancestry, and 3% African ancestry [37].

In this work, the most significantly associated variants were CFH Y402H, ARMS2 A69S, and CFB R32Q, with ORs of 6.3, 5.3, and 2.5, respectively, when the homozygous state for the risk allele was considered (Appendix 1). These results were practically unchanged in the adjusted analysis. Smoking is a major environment risk factor that is strongly and consistently associated with the development of AMD. In our study, smoking showed statistically significant differences between the groups, being more frequent in cases than in controls; this finding supports smoking as a risk factor for the development of AMD in our patients. However, when the results were adjusted by age, sex, and smoking, the OR tendencies were maintained. In our study, only patients who were diagnosed as having advanced AMD (grade 4 or 5) were included.

The results of the haplotype analysis in our sample disclosed that considering the risk variants for CFH and ARMS2 (C-T) together conferred an OR of 7, as well as an attributable risk among the exposed of 85.5% for the development of AMD (Table 3). These figures are among the highest AMD-related risk values obtained in a given population.

In our cohort, homozygosity for the C3 R102G variant was associated with a modest OR of 1.6 for AMD, which is considerably lower than the risk obtained in Caucasian populations from the United States and England [16,22], but higher than the observed risk, for example, in the French population [38]. However, a recent meta-analysis yielded a pooled OR of 1.6 for the C3 R102G variant [39], which is in agreement with our results. The R102G polymorphism generates the “fast” and “slow” electrophoretic allotypes of C3 (C3F and C3S), showing a differential capacity to bind monocyte-complement receptor C3F, which is the risk variant for AMD and has been previously reported as associated with other immune-mediated conditions [40].

The Y402H polymorphism in the CFH gene, located at 1q31, is particularly striking because of the strength of its association with late AMD. ORs greater than 5 have been consistently established for individuals homozygous for the Y402H risk allele, making this genetic association one of the strongest for a complex disorder yet to be reported [18]. Nevertheless, in African populations, it seems that an association of the Y402H risk allele with late AMD does not exist or is less marked than in other populations [30,41], indicating ethnospecific differences in AMD risk associated with particular alleles. The Q allele for the R32Q variant of CFB was shown to have a protective effect, yielding an OR of 0.3 in our study; this is in agreement with previous studies demonstrating a protective effect for this particular allele [42]. Interestingly, in our study, the protective effect of CFB R32Q was improved to 0.2 after controlling for gender, age, and smoking. However, given the low number of subjects carrying this variant in our study, this finding must be confirmed through the analysis of larger groups.  Our results support the claim that CFH, ARMS2, and to a lesser extent, CFB AMD-risk alleles are consistently associated with the disease, even in an intermixed population such as Mexican mestizos. Furthermore, the results support the notion that these alleles are the main known genetic factors for AMD development. Replication of association studies in diverse ethnic groups worldwide, especially those with a complex admixture of ancestral populations, such as Latino populations [43], would provide a better appreciation of the genetic contributions in AMD pathogenesis.

## Appendix 1. Allelic and genotype association testing results of CFH, CFB, C2, ARMS2, and C3 in AMD for cases and controls.

OR, odds ratio; CI, confidence interval. NA, Not available (the OR value couldn’t be calculated due to a zero value in a cell). To access the data, click or select the words “Appendix 1.” This will initiate the download of a compressed (pdf) archive that contains the file.

## Appendix 2. Genotypic frequencies of CFH, CFB, C2, ARMS2, and C3 in AMD cases and controls.

*Adjusted by gender, age and smoking. To access the data, click or select the words “Appendix 2.” This will initiate the download of a compressed (pdf) archive that contains the file.
